# Whole genome sequencing in oncology: using scenario drafting to explore future developments

**DOI:** 10.1186/s12885-021-08214-8

**Published:** 2021-05-01

**Authors:** Michiel van de Ven, Martijn J. H. G. Simons, Hendrik Koffijberg, Manuela A. Joore, Maarten J. IJzerman, Valesca P. Retèl, Wim H. van Harten

**Affiliations:** 1 Technical Medical Centre, University of Twente, Enschede, The Netherlands; 2 Maastricht University Medical Center, Maastricht, The Netherlands; 3 Maastricht University, Care and Public Health Research Institute (CAPHRI), Maastricht, The Netherlands; 4 University of Melbourne Centre for Cancer Research, University of Melbourne, Melbourne, Australia; 5 Peter MacCallum Cancer Centre, Melbourne, Australia; 6 Netherlands Cancer Institute-Antoni van Leeuwenhoek Hospital (NKI-AVL), Amsterdam, The Netherlands; 7 Rijnstate General Hospital, Arnhem, The Netherlands

**Keywords:** Whole genome sequencing, Implementation, Scenario drafting, Uncertainty, Oncology

## Abstract

**Background:**

In oncology, Whole Genome Sequencing (WGS) is not yet widely implemented due to uncertainties such as the required infrastructure and expertise, costs and reimbursements, and unknown pan-cancer clinical utility. Therefore, this study aimed to investigate possible future developments facilitating or impeding the use of WGS as a molecular diagnostic in oncology through scenario drafting.

**Methods:**

A four-step process was adopted for scenario drafting. First, the literature was searched for barriers and facilitators related to the implementation of WGS. Second, they were prioritized by international experts, and third, combined into coherent scenarios. Fourth, the scenarios were implemented in an online survey and their likelihood of taking place within 5 years was elicited from another group of experts. Based on the minimum, maximum, and most likely (mode) parameters, individual Program Evaluation and Review Technique (PERT) probability density functions were determined. Subsequently, individual opinions were aggregated by performing unweighted linear pooling, from which summary statistics were extracted and reported.

**Results:**

Sixty-two unique barriers and facilitators were extracted from 70 articles. Price, clinical utility, and turnaround time of WGS were ranked as the most important aspects. Nine scenarios were developed and scored on likelihood by 18 experts. The scenario about introducing WGS as a clinical diagnostic with a lower price, shorter turnaround time, and improved degree of actionability, scored the highest likelihood (median: 68.3%). Scenarios with low likelihoods and strong consensus were about better treatment responses to more actionable targets (26.1%), and the effect of centralizing WGS (24.1%).

**Conclusions:**

Based on current expert opinions, the implementation of WGS as a clinical diagnostic in oncology is heavily dependent on the price, clinical utility (both in terms of identifying actionable targets as in adding sufficient value in subsequent treatment), and turnaround time. These aspects and the optimal way of service provision are the main drivers for the implementation of WGS and should be focused on in further research. More knowledge regarding these factors is needed to inform strategic decision making regarding the implementation of WGS, which warrants support from all relevant stakeholders.

**Supplementary Information:**

The online version contains supplementary material available at 10.1186/s12885-021-08214-8.

## Contributions to the literature

- This research provides insights into what experts expect are the most important barriers and facilitators regarding the implementation of WGS as a clinical diagnostic in oncology.
- The stepwise approach to explore and quantify uncertainty used in this study can also be applied to implementation research for other health technologies that disrupt routine clinical practice.
- The findings of this study can be used to prioritize further research on the implementation of WGS.
- The drafted scenarios can be modelled in health economic evaluations to explore the impact on costs and outcomes.

## Background

Next Generation Sequencing (NGS) is used in oncology to select the optimal treatment and prevent overtreatment. Compared to single sequencing techniques, NGS is a set of techniques that sequences many genes at once. Targeted gene panels (TGP) sequence an assay of a certain number of genes. In contrast, Whole Exome Sequencing (WES) sequences all protein-coding regions of the genome and Whole Genome Sequencing (WGS) sequences, both all coding and non-coding regions of the genome. Therefore, WGS is one of the most comprehensive forms of NGS, potentially allowing more biomarkers to be identified. Although the prices of all NGS techniques have been decreasing, WGS is currently more costly [1, 2]. Even though WGS yields more genetic information compared to TGP and WES, the number of available therapies that can be prescribed based on this information remains limited [3]. However, the genetic information obtained by WGS facilitates research towards a better understanding of cancer and the discovery of new biomarkers [4], thus providing value for future patients. Consensus on the most optimal way to implement WGS in clinical practice is still lacking.

The potential of genomics to transform healthcare in several disease areas has been widely recognized, illustrated by coordinated efforts [5] towards implementation in countries worldwide [6, 7]. These are mainly focused on the organisation of care to provide WGS efficiently. So far, WGS is mostly restricted to central facilities and/or the academic setting. This means that the logistics are different from other forms of NGS, which are more frequently conducted within hospital labs. To interpret the genetic information from WGS correctly, additional expertise in bioinformatics and molecular biology is required. Thus, workforce education is another important component in implementing WGS [8–10]. Moreover, determining which subgroups of patients sufficiently benefit from WGS is needed as costs are still prohibiting sequencing at large scale.

Access to WGS for patients varies across countries. For instance, the 100,000 genomes project [11], primarily focused on cancer and rare diseases, has met its target in 2019 [12] and has been extended to sequence 300,000 genomes. In the Netherlands, WGS is only accessible for cancer patients through enrolment in the “Center for Personalized Cancer Treatment (CPCT-02)” or “WGS Implementation in the standard Diagnostics for Every cancer patient (WIDE)” studies. In general, WGS is primarily being used in the clinical research setting, while implementation into clinical practice is currently limited. The Technology Assessment of Next Generation Sequencing in Personalized Oncology (TANGO) study investigates the value of WGS for clinical diagnostics compared to other NGS techniques in the Netherlands [13]. The current study was conducted from this perspective, by drafting scenarios as part of the Health Technology Assessment.

Scenario drafting makes possible future pathways more explicit [14], thus leading to a better understanding of important uncertainties [15] and improved ability to anticipate future changes. Scenarios are drafted through an iterative process, starting with a literature search, followed by several expert discussions on potential future developments [16]. Scenarios are coherent stories that describe deviations from the current situation. They are not meant as predictive, but they are a useful tool to explore possible futures [17]. Scenario drafting is often used in environmental and management sciences [16], while its application and that of similar approaches in healthcare is limited [18–20]. Scenarios can be quantified by using expert elicitation to parametrize unknown variables. Subsequently, these scenarios can be used to inform model-based analyses [18], thereby quantifying the consequences of the scenarios.

The key objective of this study is to draft scenarios that reflect several different possible future pathways for the implementation of WGS into clinical practice in oncology. Subsequently, the likelihood that each of these scenarios will occur within a time horizon of 5 years will be estimated using expert elicitation.

## Methods

**Fig. 1:**
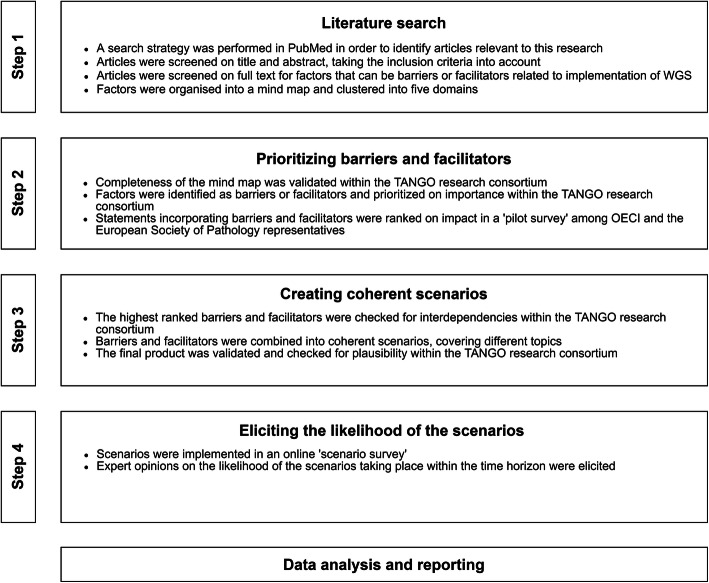
Flowchart of the used methodology for creating and eliciting the probability of the scenarios. WGS, whole genome sequencing; TANGO, Technology assessment of next generation sequencing for personalized oncology; OECI, Organisation of European Cancer Institutes

A four-step process was adopted for scenario drafting: “literature search”, “prioritizing barriers and facilitators”, “creating coherent scenarios”, and “eliciting the likelihood of the scenarios.” Within these steps, validation and plausibility checks with international experts were included. An overview is displayed in Fig. 1. Barriers and facilitators are factors that can either have an impeding or facilitating role in the implementation of WGS.

### Step 1: literature search

PubMed was searched for literature, using MeSH-terms and free text words. The full detailed search strategy is listed in the Additional file 1: Appendix I. Studies were included that described barriers and facilitators related to the implementation of complex and disruptive technologies in general and of WGS as a clinical diagnostic in particular. The articles found by the search strategy were screened on title and abstract by two authors (MV, MS), taking the inclusion criteria into account. Subsequently, the remaining articles were screened on full text for factors that may be barriers or facilitators in the implementation of WGS. The identified factors were summarized under common headers and organized into a mind map. The factors were clustered into five domains*: ‘clinical utility and evidence generation’, technical’, ‘reimbursement’, ‘social’,* and *‘market access’* [19]. In a research consortium session, we verified that no important factors were missing. The TANGO consortium comprised of experts within the field of oncology, pathology, genetics, informatics, health economics, health technology assessment, legislation, ethics, and of patient representatives.

### Step 2: prioritizing barriers and facilitators

We identified the factors as barriers or facilitators and prioritized them in an interactive session with our research consortium. Additionally, statements that incorporate barriers and facilitators were ranked on their potential impact on the implementation of WGS in a questionnaire, further called ‘pilot survey’, among 14 representatives from the Organisation of European Cancer Institutes (OECI) and the European Society of Pathology. These representatives included pathologists, oncologists, pulmonologists, clinical scientists based in Croatia, Denmark, Italy, the Netherlands, Portugal, Moldova, Russia, Switzerland, Turkey, and the United Kingdom. Seven statements were ranked from most to least important by each representative. The statement that was ranked as most important would receive seven points, and the statement that was ranked as least important, one point. The final ranking was made by tallying the awarded points across representatives.

### Step 3: creating coherent scenarios

Barriers and facilitators that were ranked highest in the pilot survey were used to develop coherent scenarios. The principles of Cross Impact Analysis [21] were used to create coherent scenarios that include multiple interdependent developments or consequences. Possible interdependencies between barriers and facilitators were considered by consulting the experts within our research consortium. The reasoning behind creating scenarios with multiple interdependent barriers and facilitators is that the future developments and their consequences are most likely related. Therefore, it would lead to bias if interdependent factors would be viewed in isolation. Subsequently, barriers and facilitators were combined in scenarios so that they cover several topics related to the implementation and cost-effectiveness of WGS. Each scenario had a similar structure: one possible future development followed by two or three consequences of that development.

#### Validation

The final product of the scenarios was validated and checked for plausibility by discussing its content with the experts within the TANGO research consortium. Additionally, the scenarios were checked on ambiguity in language.

### Step 4: eliciting the likelihood of the scenarios

The scenarios were implemented in an online survey, using Qualtrics^XM^ [22], further called the ‘scenario survey.’ The target population was international experts with expertise of genomics or related fields, as well as patients that may be affected by the use of WGS.

**Fig. 2:**
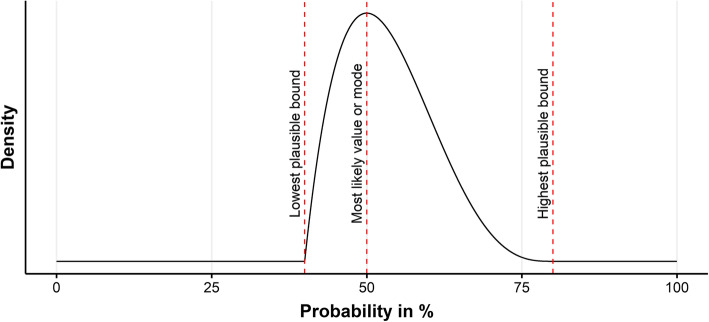
Example of the values elicited in the scenario survey related to the PERT distribution. In this example, the lowest plausible bound equals 40%, most likely value or mode equals 50%, and highest plausible bound equals 80%

The current situation in practice, i.e. the status quo, was presented in the scenario survey as the framework from which the scenarios deviated. Experts were asked for their opinion on the likelihood of the development and consequences taking place within the time horizon. Furthermore, the likelihood that the entire scenario, meaning both the development and its consequences, would occur within the time horizon was elicited. Three probabilities were elicited for scoring a likelihood: the mode or most likely probability that the development may occur; the lowest plausible bound where it would be extremely implausible that the real probability was below this number; and the highest plausible bound where it would be extremely implausible that the real probability was above this number. An example is displayed in Fig. 2. Each elicited likelihood could be scored between 0% (extremely unlikely) and 100% (extremely likely). Eliciting the mode as well as the lower and upper bounds provided a measure of uncertainty at the individual level and was based on the Sheffield elicitation framework [23]. While no calibration questions were used, experts could skip a scenario if it was beyond the scope of their expertise. The survey was anonymised, and experts were asked for informed consent beforehand. The scenario survey was distributed among the authors’ professional networks using (social) media channels.

### Data analysis

Based on the elicited probabilities, individual Program Evaluation and Review Technique (PERT) probability density functions (PDF) were determined. In addition to a point estimate, this approach provides a measure of uncertainty at the individual level. The PERT distribution is a modified beta distribution [24] and is defined by three parameters: a minimum, maximum, and most likely (mode). Subsequently, to aggregate individual opinions, we performed unweighted linear opinion pooling by taking 50,000 random samples from each individual PERT PDF. The combined random samples from all experts were visualized using kernel density estimation. The benefit of this nonparametric approach is that it can visualize the consensus, or lack thereof, among experts. The mean, median, and the highest density intervals (HDI) for the 80th percentile of these linear pools were extracted and reported. HDI is the narrowest possible interval that covers a given amount of density and therefore provides insight into how uncertain the group of experts is about the likelihood of a scenario. We have classified questions that have an 80% HDI bandwidth below or equal to 50, to have a relatively strong consensus. In comparison, an 80% HDI bandwidth larger than 50 indicates a relatively weak consensus among experts. The 80% HDI bandwidth is calculated by subtracting the 80% HDI lower bound from the 80% HDI upper bound. Data analyses were performed in R statistical software [25]. The R-code of the data analysis is provided in a supplementary file.

## Results

### Step 1: literature search

**Fig. 3:**
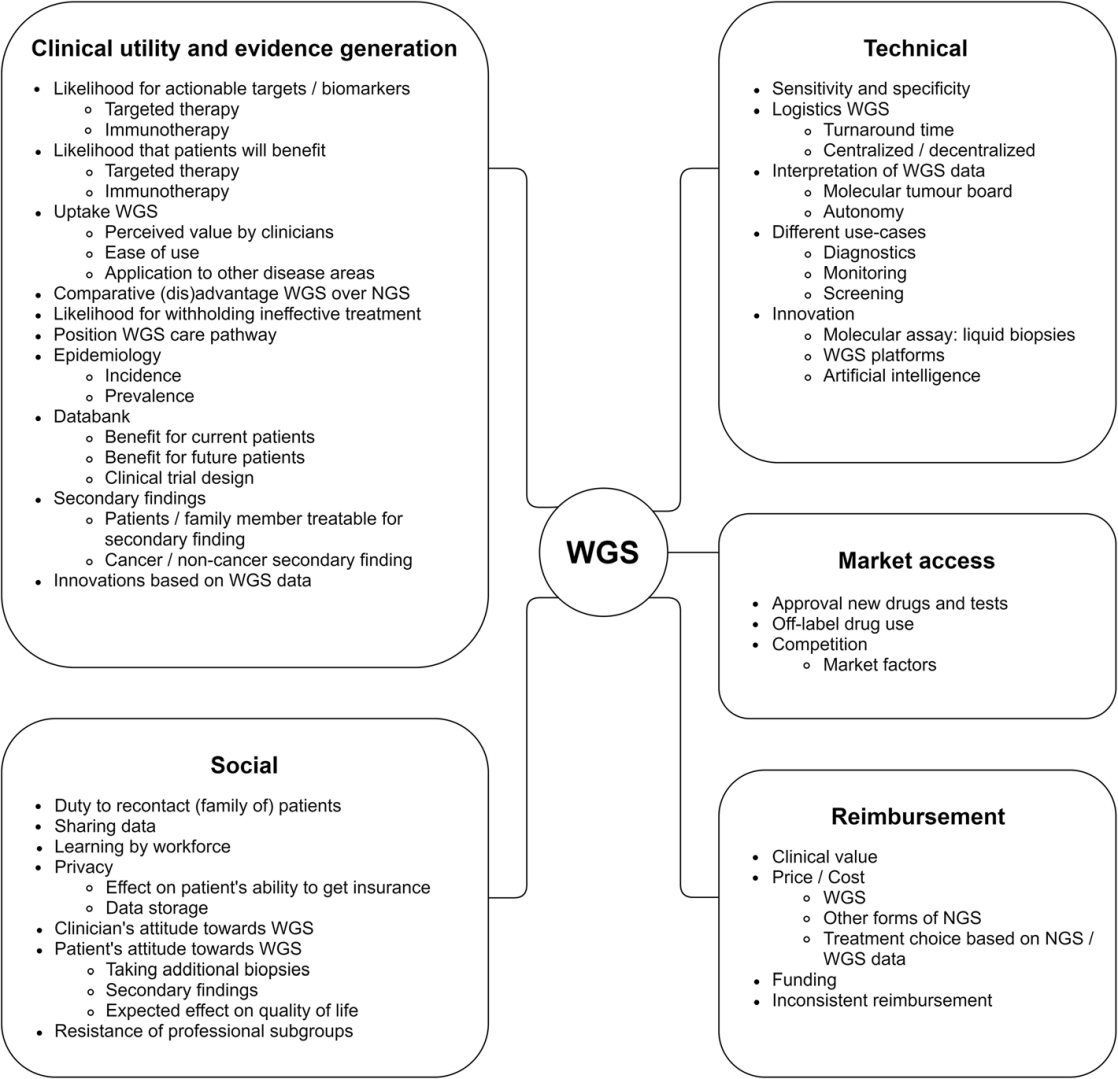
Factors identified with the literature search, stratified per domain. WGS, Whole Genome Sequencing; NGS, Next Generation Sequencing

The literature search includes articles up to June 2019. The search strategy resulted in 111 articles, of which 41 were excluded based on title and abstract. The remaining 70 articles were screened on full text. One hundred ninety-two factors were identified after screening the full texts and were summarized under 62 common headers, which are displayed in Fig. 3. These factors were clustered into the domains: clinical utility and evidence generation (*n* = 24), technical (*n* = 15), reimbursement (*n* = 7), social (*n* = 12), and market access (*n* = 4). More details on the literature search are provided in the Additional file 1: Appendix II.

### Step 2: prioritizing barriers and facilitators

The barriers and facilitators that were prioritized from most to least important by experts in the pilot survey, are listed in Table 1.

**Table 1:** Ranking of barriers and facilitators, results from the pilot survey

Rank	Barriers	Facilitators
1	The clinical utility of WGS compared to TGPs will not be demonstrated sufficiently.	The clinical utility of WGS compared to TGPs has been demonstrated sufficiently.
2	The turnaround time of WGS will remain significantly longer compared with that of TGPs.	WGS will be included in basic health insurance.
3	The price of WGS will remain too high.	The price of WGS will drop significantly.
4	A technology that is superior in terms of cost and/or clinical utility compared to WGS will become available.	The interpretation of WGS results will become as easy as TGP results.
5	The interpretation of WGS results will not become easier.	The turnaround time of WGS will decrease and become equal to that of TGPs.
6	Fresh frozen biopsies will remain the only reliable source of DNA for WGS.	Other type of biopsies can be used for WGS, for example, liquid biopsies and FFPE biopsies.
7	WGS will not become part of basic health insurance.	No other technology that would compete with WGS will become available.

The ranked barriers and facilitators are ordered from most important to least important

*WGS* Whole Genome Sequencing, *TGP* Targeted Gene Panel, *DNA* deoxyribonucleic acid, *FFPE* Formalin-Fixed Paraffin-Embedded

### Step 3: creating coherent scenarios

A full description of the status quo and scenarios are listed in the Additional file 1: Appendix III. Nine scenarios were created and are listed in Table 2. These scenarios were labelled as: ‘innovation in WGS devices’ (scenario 1); ‘the discovery of a new actionable biomarker for immunotherapy’ (scenario 2); ‘the effect of centralizing WGS’ (scenario 3); ‘introducing WGS as a clinical diagnostic in oncology’ (scenario 4); ‘a new competing NGS panel ‘X” (scenario 5); ‘technical performance’ (scenario 6); ‘approval of new drugs for new actionable targets’ (scenario 7); ‘approval for off-label drug prescription’ (scenario 8); and ‘better treatment response to actionable targets found by WGS’ (scenario 9).

**Table 2:** Scored likelihoods of the linear pooled estimates

Scenario questions (Q)	Brief description	Experts (n)	Mean	Median	80% HDI	80% HDI bandwidth
**Scenario 1**	**Innovation in WGS devices**					
Q1	WGS testing kit with 50% cheaper initial investment costs	18	65.5	69.2	51.5–100.0	48.5
Q2	Interpretation MTB only required for 5% of the patients	17	38.8	31.6	1.8–68.9	67.1
Q3	Average turnaround time reduced to 7 days	17	54.2	63.4	17.6–98.1	80.5
**Q4**	**Overall scenario taking place within the next 5 years**	**16**	**46.0**	**52.1**	**0.1–85.5**	**85.4**
**Scenario 2**	**The discovery of a new actionable biomarker for immunotherapy**					
Q1	WGS is the only technique that can identify new biomarkers	17	28.3	21.8	0.0–49.0	49.0
Q2	WGS detects new biomarker for immunotherapy in 20% of the patients	17	46.9	48.4	11.6–90.2	78.6
Q3	90% of the physicians offer WGS to patients	16	65.5	72.1	43.7–98.0	54.3
Q4	90% of patients prefer WGS to other molecular diagnostics	15	66.7	80.3	25.9–99.3	73.4
**Q5**	**Overall scenario taking place within the next 5 years**	**17**	**45.3**	**45.5**	**0.3–81.3**	**81.0**
**Scenario 3**	**The effects of centralizing WGS**					
Q1	Centralizing WGS leads to large reduction costs and turnaround time	16	52.5	51.4	19.3–88.8	69.5
Q2	Costs WGS decreased to €1000.- per patient	16	54.9	54.9	30.7–85.6	54.9
Q3	Turnaround time WGS decreased to 5 days	16	37.9	29.9	0.0–69.5	69.5
Q4	All hospitals will adopt WGS	15	58.7	68.7	24.1–97.1	73.0
**Q5**	**Overall scenario taking place within the next 5 years**	**15**	**26.5**	**24.1**	**0.0–45.1**	**45.1**
**Scenario 4**	**Introducing WGS as a clinical diagnostic**					
Q1	WGS available as standard diagnostic test in clinical practice	17	64.5	76.1	31.6–99.9	68.3
Q2	WGS detects actionable target (targeted therapy) in 12% of the patients	17	68.8	74.7	55.3–100.0	44.7
Q3	Turnaround time WGS decreased to 14 days	17	76.1	80.3	61.2–99.8	38.6
Q4	Costs WGS decreased to €3000.- per patient	16	81.1	83.6	69.7–99.8	30.1
Q5	WGS will be used instead of standard diagnostics	17	58.7	65.7	23.2–95.9	72.7
**Q6**	**Overall scenario taking place within next 5 years**	**17**	**55.3**	**68.3**	**15.5–99.0**	**83.5**
**Scenario 5**	**A new competing NGS panel ‘X’**					
Q1	New liquid NGS panel ‘X’ enters the market	16	67.1	75.7	45.0–100.0	55.0
Q2	NGS panel ‘X’ detects actionable targets in 8% of the patients	15	66.6	77.4	46.2–95.2	49.0
Q3	Less invasive liquid biopsies can be used for NGS panel ‘X’	15	56.1	59.9	16.7–88.0	71.3
Q4	Turnaround time NGS panel ‘X’ is on average 2 days	15	48.5	51.9	0.0–74.5	74.5
Q5	Costs NGS panel ‘X’ are €300.- per patient	15	51.6	51.6	18.4–93.4	75.0
Q6	NGS panel ‘X’ will be used instead of WGS	16	56.3	62.4	21.6–94.2	72.6
**Q7**	**Overall scenario taking place within the next 5 years**	**15**	**40.8**	**39.8**	**0.0–78.1**	**78.1**
**Scenario 6**	**Technical performance**					
Q1	Success rate tissue biopsies and sequencing process of WGS improve	15	59.0	64.7	22.9–86.1	63.2
Q2	Tissue biopsies successfully taken in 80% of the patients	15	55.1	58.5	20.2–96.9	76.7
Q3	Sequencing process of WGS successful in 95% of the patients	14	50.7	59.6	0.0–73.3	73.3
Q4	More than 80% of the patients sequenced successful	14	52.7	58.4	18.5–89.9	71.4
Q5	Costs WGS stay fixed at €4500.- per patient	14	47.0	47.3	22.8–80.0	57.2
**Q6**	**Overall scenario taking place within the next 5 years**	**15**	**40.0**	**39.2**	**0.0–69.7**	**69.7**
**Scenario 7**	**Approval of new drugs for new actionable targets**					
Q1	Approval new targeted therapies for new targets discovered by WGS	14	55.0	54.6	26.1–97.8	71.7
Q2	New actionable targets can only be detected by WGS	15	34.6	27.9	0.0–56.2	56.2
Q3	WGS detects new biomarker for targeted therapy in 20% of the patients	15	41.5	44.4	0.0–62.8	62.8
Q4	90% of the physicians prefer using WGS as molecular diagnostic	14	66.8	71.4	53.6–95.2	41.6
Q5	90% of patients prefer to receive WGS as molecular diagnostics	14	68.6	78.6	28.4–98.7	70.3
**Q6**	**Overall scenario taking place within the next 5 years**	**14**	**35.5**	**28.1**	**0.0–69.8**	**69.8**
**Scenario 8**	**Approval for off-label drug prescription**					
Q1	Off-label drug use will be allowed based on research on WGS data	15	65.6	66.9	39.5–99.7	60.2
Q2	Off-label drug prescription only allowed for targets found by WGS	14	47.9	42.0	6.3–92.0	85.7
Q3	WGS detects actionable target for off-label targeted therapy in 5% of the patients	14	60.4	73.1	17.8–89.8	72.0
Q4	95% of the physicians prefer using WGS as molecular diagnostic	15	72.1	83.6	43.9–98.8	54.9
Q5	All patients prefer to receive WGS as molecular diagnostics	14	69.5	85.2	36.6–99.5	62.9
**Q6**	**Overall scenario taking place within the next 5 years**	**14**	**47.3**	**43.9**	**25.2–92.3**	**67.1**
**Scenario 9**	**Better response to actionable targets found by WGS**					
Q1	Better treatment response in patients with targets identified with WGS	14	18.5	9.3	0.0–39.7	39.7
Q2	Treatment response targeted therapy increased to 10%	16	35.7	24.0	0.0–73.7	73.7
Q3	WGS detects biomarkers that are better predictors for treatment response	14	42.5	48.6	0.0–64.7	64.7
Q4	All physicians prefer using WGS as molecular diagnostic	16	54.6	60.3	13.1–96.4	83.3
Q5	All patients prefer to receive WGS as molecular diagnostics	16	55.5	60.5	15.9–96.9	81.0
**Q6**	**Overall scenario taking place within the next 5 years**	**15**	**25.7**	**26.1**	**0.0–42.3**	**42.3**

*80% HDI* 80% Highest Density Interval, *WGS* Whole Genome Sequencing, *MTB* Molecular Tumour Board, *NGS* Next Generation Sequencing

### Step 4: likelihood of the scenarios

Twenty-two international experts responded to the scenario survey of whom 19 completed the survey, 1 expert did not fill in any question, and 2 experts wished not to participate. The scenario survey was completed by experts within the field of oncology, genetics, informatics, pathology, health economics, health technology assessment, pulmonary disease and lung cancer, who resided in the Netherlands, Australia, Denmark, and Singapore. One expert completed the survey in a different way than was statistically intended and was removed from the quantitative analysis. More details are listed in Additional file 1: Appendix IV.

**Fig. 4:**
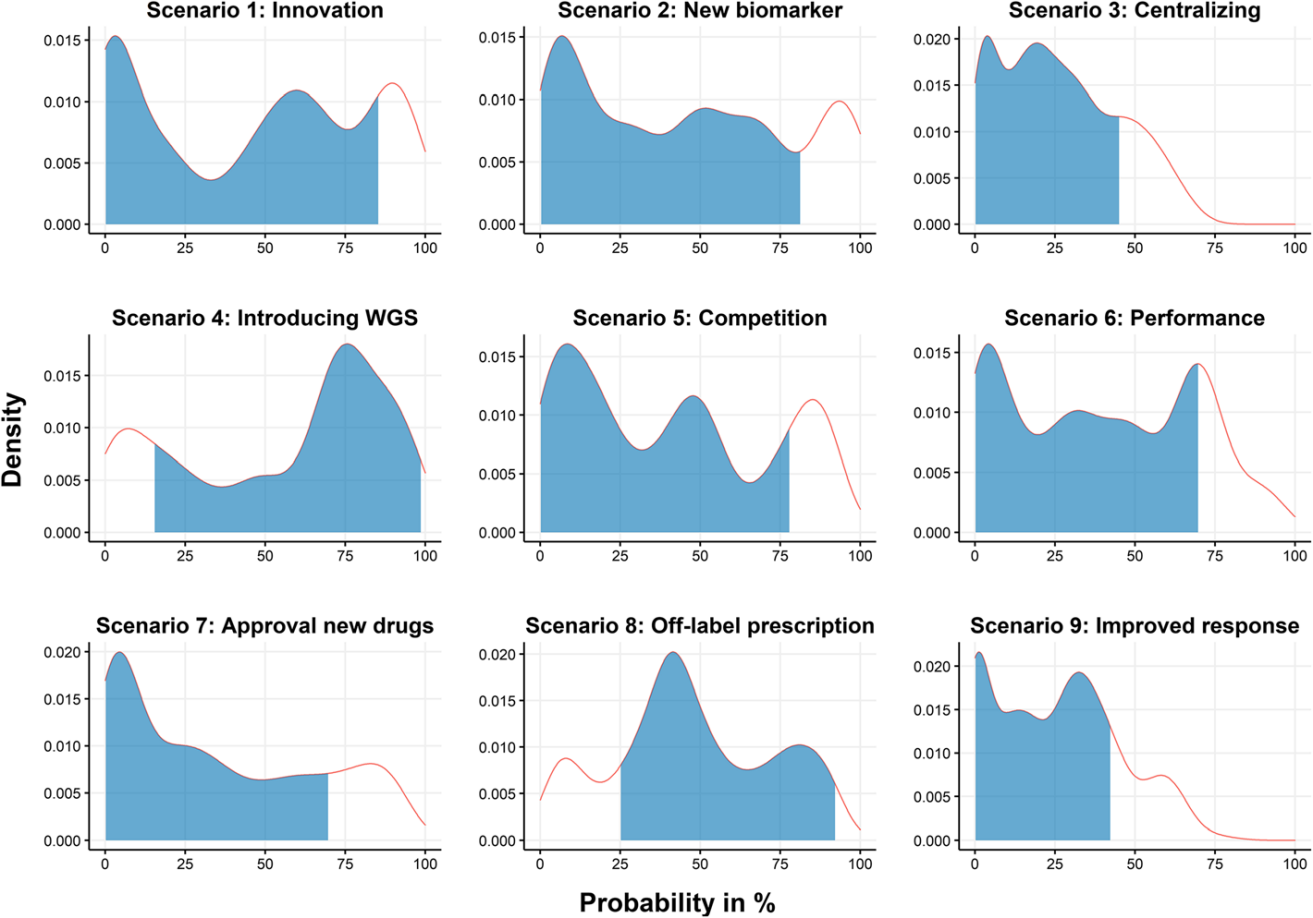
Linear pools of individual PERT distributions for the overall likelihood of each scenario. The blue-shaded area under the curve represents the 80% highest density interval. The scenarios concerned: ‘innovation in WGS devices’ (scenario 1); ‘the discovery of a new actionable biomarker for immunotherapy’ (scenario 2); ‘the effect of centralizing WGS’ (scenario 3); ‘introducing WGS as a clinical diagnostic in oncology’ (scenario 4); ‘a new competing NGS panel ‘X” (scenario 5); ‘technical performance’ (scenario 6); ‘approval of new drugs for new actionable targets’ (scenario 7); ‘approval for off-label drug prescription’ (scenario 8); and ‘better treatment response to actionable targets found by WGS’ (scenario 9)

The results of the scenario survey are listed in Table 2. Figure 4 depicts the linear opinion pools of the overall likelihood of each scenario. Differences in opinion among experts are reflected in the observed multimodality in the linear opinion pools. There was a relatively weak consensus on most overall scenarios. Therefore, we also report on some of the sub-scenarios that had a relatively strong consensus.

Based on the median, the scenario concerning ‘the introduction of WGS as a clinical diagnostic’ (scenario 4) had the highest likelihood, but with a relatively weak consensus (median: 68.3%, [80% HDI: 15.5–99.0]). Within this scenario, there was a relatively strong consensus on the likelihoods that: ‘WGS will detect more actionable targets than current standard diagnostics (74.7%, [55.3–100.0)’; ‘the turnaround time will decrease to fourteen days (80.3%, [61.2–99.8])’; and ‘the costs will decrease to €3,000 per patient (83.6%, [69.7–99.8])’.

The scenario concerning ‘innovations in WGS devices’ (scenario 1) had the second-highest likelihood, but also with a relatively weak consensus (52.1%, [0.1–85.5]). Within this scenario, there was only a relatively strong consensus on the likelihood of ‘the development of a new WGS testing kit that is 50% cheaper in initial investment costs (69.2%, [51.5–100.0])’.

The scenario concerning ‘the discovery of a new actionable biomarker for immunotherapy’ (scenario 2) had the third-highest overall likelihood and had a relatively weak consensus (45.5%, [0.3–81.3]). Within this scenario, there was only a relatively strong consensus on the likelihood that ‘WGS is the only technique that can identify new biomarkers (21.8%, [0.0–49.0])’.

The scenario concerning ‘a new competing NGS panel ‘X” (scenario 5) had the fourth-highest overall likelihood and had a relatively weak consensus (39.8%, [0.0–78.1]). Within this scenario, there was only a relatively strong consensus on the likelihood that ‘NGS panel ‘X’ detects actionable targets in 8% of the patients (77.4%, [46.2–95.2])’.

The scenario concerning ‘the approval of new drugs for new actionable targets’ (scenario 7) had the third-lowest likelihood, with relatively weak consensus (28.1%, [0.0–69.8]). Within this scenario, there was only a relatively strong consensus on the likelihood that ‘90% of the physicians prefer using WGS as a molecular diagnostic (71.4%, [53.6–95.2])’.

The scenario concerning ‘better response to actionable targets found by WGS’ (scenario 9) had the second-lowest likelihood, with a relatively strong consensus (26.1%, [0.0–42.3]). Within this scenario, there was also a relatively strong consensus about the likelihood of ‘a better treatment response in patients with targets identified with WGS (9.3%, [0.0–39.7])’.

The scenario concerning ‘the effect of centralizing WGS’ (scenario 3) had the lowest likelihood, with a relatively strong consensus (24.1%, [0.0–45.1]).

## Discussion

This study aimed to investigate possible future developments facilitating or impeding the use of WGS by means of scenario drafting. Based on our literature review, we identified 62 unique barriers and facilitators for the implementation of WGS. Price, clinical utility, and turnaround time were considered as most essential for the implementation of WGS. We created nine coherent scenarios covering different pathways for the implementation of WGS into clinical practice in oncology, by combining various aspects and parameters. The scenario in which WGS would be introduced as a clinical diagnostic (scenario 4) had the highest likelihood of taking place within the next 5 years with a relatively weak consensus (68.3%, [15.5–99.0]). The scenarios about a better treatment response to actionable targets that were found with WGS (scenario 9) and the centralization of organizing WGS (scenario 3) had the lowest likelihoods, with a relatively strong consensus (26.1%, [0.0–42.3] and 24.1%, [0.0–45.1], respectively).

The factors that were found in the literature search span several different domains. It implies that, even if one barrier is overcome, other barriers may still prevent widespread use of WGS. For example, if the clinical utility of WGS is clearly established, barriers in the social domain may hinder the use of WGS. Therefore, a strategy to responsibly introduce WGS would be most effective if multiple or all these domains are considered.

Ranking the barriers and facilitators in order of importance could assist with selecting those that should receive the most attention. Most important seems to address the unknown clinical utility of WGS compared to other NGS techniques. The unknown or unclear benefit to patients has been identified earlier, as a common problem in the implementation of healthcare technologies [26]. Additionally, being able to demonstrate the added value of a technology is often the basis for reimbursement, thereby increasing the rate of diffusion [27]. However, the scenario concerning a better treatment response to actionable targets identified by WGS (scenario 9) was with a relatively strong consensus, deemed unlikely by experts to take place within the foreseeable future. Other scenarios describing the potential clinical value of WGS were also deemed unlikely but with widely varying opinions. This concerned for instance the chance of discovering a new biomarker for immunotherapy that can be found by WGS (scenario 2), or the discovery of new actionable targets based on WGS data for which new targeted treatments will become available (scenario 7). This means that with current knowledge it is not very likely that WGS will receive reimbursement for use in the clinical practice, limiting the use of WGS to clinical research for the foreseeable future.

Furthermore, the results related to the scenario in which WGS was introduced as a clinical diagnostic (scenario 4) show that most experts find it relatively likely, with a relatively strong consensus that within 5 years costs of WGS will have decreased to 3000 euros per patient. This coincides with a previous study analysing the potential developments in the costs of WGS [2]. Additionally, experts deem it rather likely that the turnaround time will have decreased to 14 days. Even so, there is little consensus among experts whether those reductions would mean that WGS would be used instead of current standard diagnostics. Apparently, either the reductions in costs and turnaround time are not substantial enough to warrant the use of WGS, or other factors play a more dominant role in the decision to use WGS instead of current standard diagnostics. Although these other factors were not included in the scenario, Table 1 provides evidence that the clinical utility plays a significant role in the implementation of WGS. In scenario 4, the clinical utility of WGS remains unchanged relative to the base-case, which may be the reason that the consensus among experts is not stronger.

A strength of this study is that we included a diverse group of international experts in multiple steps of scenario drafting. While our approach does not guarantee that important barriers and facilitators were not missed, involving a diverse group of experts minimizes the likelihood that important barriers and facilitators were missed, while it also provides a diverse range of opinions. This is especially important in a field as complex and fast-moving as molecular oncology. An additional strength is that our approach of scoring likelihoods allowed us to estimate uncertainty at both the individual and group levels. Unlike in a stepwise, Delphi-like approach where the goal is to reach consensus in a group discussion, we were able to quantify the degree of consensus among the participating experts.

A limitation of this study is that the degree of consensus or uncertainty among experts for the overall likelihood is relatively large for most scenarios. This can have multiple causes. First, it may have been challenging to quantify and score the scenarios as we noticed that experts find difficulty in giving a quantitative estimate when evidence is lacking. Second, future developments of technologies like WGS may just be too inherently difficult to predict. Third, the sample size could have been too small. However, it is not very likely that increasing the sample size would have in fact reduced uncertainty. Fourth, the cognitive burden imposed by the scenarios may have been too high. This is a common issue with scenarios that are based on the principles of Cross Impact Analysis [28]. An attempt was made to limit the cognitive burden of the scenarios by limiting the number of included barriers or facilitators. Simplifying the scenarios can be challenging, given that the scenarios need to remain internally valid.

The scores of the scenarios give a clear view on what experts think is likely and what they agree and disagree on regarding the implementation of WGS. This information can be used to give direction to policy and future research about WGS to reduce this lack of knowledge and thus uncertainty. This is important since WGS is deemed likely to be implemented as clinical diagnostic in oncology within the upcoming years.

Future research should be focussed on investigating what clinical benefits WGS potentially has to offer and when it will have been demonstrated sufficiently. Even though the respondents in our study found it relatively unlikely that response will be better to actionable targets found by WGS, the clinical utility can be increased by, among others, approving more treatment for off-label use and the discovery of novel biomarkers that can be identified with WGS. However, this is a very fast-moving field, so statements on expected time frames in the scenarios have to be interpreted in the correct context. Establishing a clear clinical benefit can also have consequences for other barriers and facilitators, such as the reimbursement status of WGS. Research on making WGS as a technique cheaper and faster to perform, will also contribute to its implementation in clinical practice. Additionally, WGS may provide value through other types of utility beyond clinical utility, such as personal utility. Establishing how personal utility can contribute towards the implementation of WGS might also be an exciting avenue for future research.

## Conclusion

Based on current expert opinions, the implementation of WGS as a clinical diagnostic in oncology depends heavily on the price, clinical utility (both in terms of identifying actionable targets as in adding sufficient value in subsequent treatment), and turnaround time. These aspects and the optimal way of service provision are the main drivers for the implementation of WGS and should be focused on in further research. More knowledge regarding these factors is needed to inform strategic decision making regarding the implementation of WGS, which warrants support from all relevant stakeholders.

## Supplementary Information

**Additional file 1.** Appendix.

**Additional file 2.** R code used in the analysis.

## Data Availability

The datasets generated and analysed during the current study are freely available via 10.5281/zenodo.4650466 [29] in the Zenodo repository [30].

## Abbreviations

WGS: Whole Genome Sequencing
NGS: Next Generation Sequencing
TGP: Targeted Gene Panel
WES: Whole Exome Sequencing
CPCT: Center for Personalized Cancer Treatment
WIDE: WGS Implementation in the standard Diagnostics for Every cancer patient
TANGO: Technology Assessment of Next Generation Sequencing in Personalized Oncology
OECI: Organisation of European Cancer Institutes
PERT: Program Evaluation and Review Technique
PDF: Probability Density Function
HDI: Highest Density Interval
FFPE: Formalin-Fixed Paraffin-Embedded
