# Supplementary material for: Whole genome sequencing in oncology: using scenario drafting to explore future developments
Source: BMC Cancer. 2021 May 1;21:488. doi: 10.1186/s12885-021-08214-8 (PMC8088550; doi:10.1186/s12885-021-08214-8)
Supplement: Supplementary file 1 — Additional file 1. Appendix. [file 12885_2021_8214_MOESM1_ESM.docx]

# Appendix I. Literature search terms

Literature was searched using the search strategy that is listed in table A1. The following search terms were used: ‘advanced cancer’, ‘metastatic cancer’, ‘Non-small cell lung cancer’, ‘disruptive technology’, ‘innovation’, ‘scenario drafting’, ‘future scenario’, ‘implementation’, ‘whole genome sequencing’, ‘next generation sequencing’, ‘molecular diagnostic’, ‘clinical diagnostic’, ‘personalised medicine’. These search terms were incorporated in the search strategy, using MeSH-terms, synonyms, and truncations in combination with Boolean operators (‘AND’ and ‘OR’).

Table A1 search strategy

| **Search** | **Query** | **Hits** |
| --- | --- | --- |
|  | **Patient** |  |
| #1 | (Neoplasm Metastasis[MESH]) OR (advanced cancer[tiab]) OR (metastatic cancer[tiab]) | 217,931 |
| #2 | (Carcinoma, Non-Small-Cell Lung[MESH]) OR (non-small cell lung cancer[tiab]) | 71,427 |
| **#3** | **#1 OR #2** | **283,465** |
|  | **Intervention** |  |
| #4 | (Disruptive Technology[MESH]) OR (Disruptive Technology[tiab]) | 235 |
| #5 | (Diffusion of Innovation[MESH]) OR (innovation[tiab]) | 49,947 |
| #6 | (Forecasting[MESH]) OR (scenario drafting[tiab]) OR (scenario creation[tiab]) OR (future scenario*[tiab]) | 85,753 |
| #7 | (Implementation[tiab]) | 243,124 |
| **#8** | **#4 OR #5 OR #6 OR #7** | **370,393** |
|  | **Control** |  |
| #9 | (whole genome sequencing[MESH]) OR (whole genome sequencing[tiab]) OR (next generation sequencing[tiab]) | 50,998 |
| #10 | (Pathology, Molecular[MESH]) OR (molecular diagnostic[tiab]) OR (Diagnostic Test Approval[MESH]) OR (clinical diagnostic[tiab]) OR (Genetic Testing[MESH]) OR (genetic test*[tiab]) OR (molecular test*[tiab]) | 80,018 |
| #11 | (Precision Medicine[MESH]) OR (personalised medicine[tiab]) | 18,428 |
| **#12** | **#9 OR #10 OR #11** | **143,684** |
|  | **Patient & Intervention & Control** |  |
| **#13** | **#3 AND #8 AND #12** | **111** |
|  | | |

# Appendix II. Flowchart of the literature search including the extracted factors that were used for scenario drafting

The literature search includes articles up to June 2019. The flowchart of the literature search is displayed in figure A1. From the 66 resulting articles, 192 factors were extracted by reading the full text. Many of these factors were synonyms from one another or different descriptions of the same thing. Therefore, we were able to summarize these factors under 62 common headers and clustered them into the domains: clinical utility and evidence generation (n=24), technical (n=15), reimbursement (n=7), social (n=12), and market access (n=4). The original 192 factors are listed in table A2.


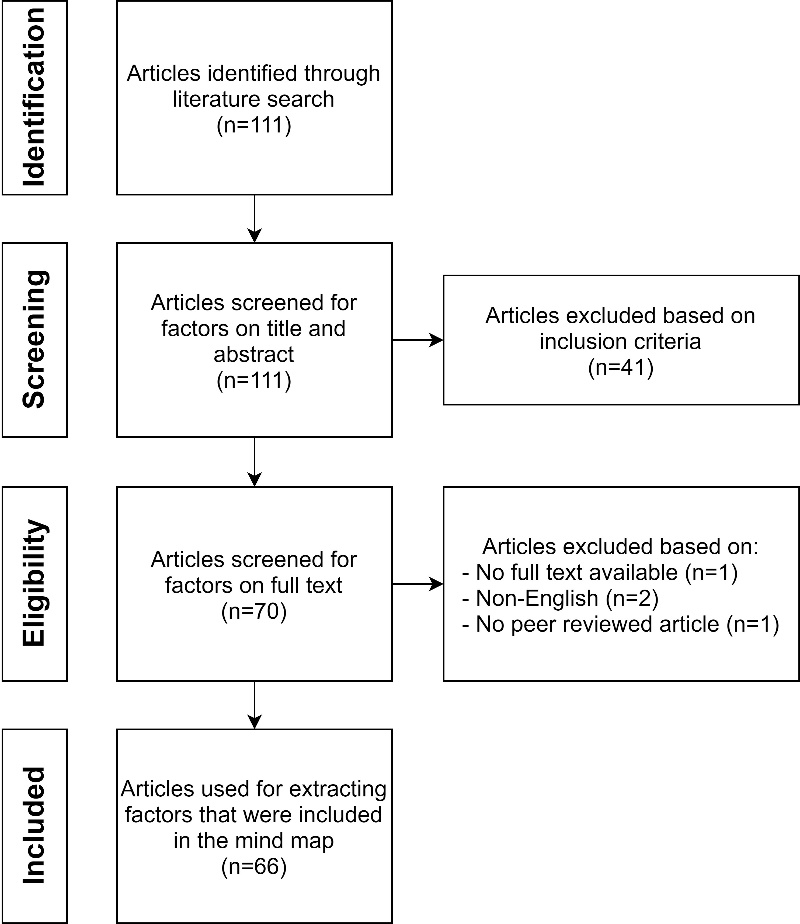


Figure A1. Flowchart of the literature search

Table A2. Factors extracted from literature clustered into the domains

| **Domain (*)** | **Factors extracted from literature** |
| --- | --- |
| **Clinical utility and evidence generation (n=76)** | Actionable genetic variants; Biomarkers; Genomic alterations in lung, Adenocarcinoma; Identification of driver mutations; Immunotherapies; Molecular pathway; Tailoring treatment; Targeted therapies; Targeted therapy in cancer; Chromosomal aberration; Tumour mutational burden, micro satellite instability, mismatch repair; Patient heterogeneity; Challenges of treatment of NSCLC; Challenges of effective diagnosis and predictive analysis; Clinical benefits; Efficacy; Patient selection; Pharmacogenetics; Pharmacogenomic potential in advanced cancer patients; Selecting patients who benefit most; Tumour heterogeneity; Unclear survival benefits; Detection rate; Translational research; Implementation of molecular diagnostics; Reliability of subtyping in NSCLC; Validation; Validity; Quality assurance; Quality indicators; Cancer dynamics; Differences in biomarker tests; Differences in quality of care; Differences in tests; Analytical and clinical validity; Adjustment of treatment  decision support; Optimal treatment approach; Optimal treatment strategies;  optimized testing strategies; Treatment resistance; The possibility of clinical sequencing in the management of cancer; Need for developing biomarkers to optimise drug development and clinical use; DNA and transcriptome data integration; Big data; Biobanking; Bioinformatic analysis; Biomarker discovery  biomarker driven clinical trials; Clinical trial implementation and feasibility; Trial recruitment; Clinical trial design; Clinical trial enrolment based on genomic testing; Complex data; Complex molecular landscape; Comprehensive genomic profiling; Data interpretation; Development of new therapies through biomarker-driven clinical trials; Development of novel drugs and the implementation of precision medicine; Larger clinical trials; Larger trials; New actionable targets; New biomarkers; New drugs; New molecular targets and biomarkers; New targeted therapies; Novel targets and therapeutics; Novel therapies; Novel trial design; Patient access to trials; Patient participation in clinical trials; Sequencing data; Standardized patient databases; Trial designs; Delays; Unsolicited findings. |
| **Technical (n=59)** | Sensitivity; Sensitivity / specificity; Success rate; Biopsy often infeasible; Appropriate tissue samples; Failure rate; False positives; Improved test performance; Test discordance; Re-biopsy; Sample quality; Test prioritization; Centralization; Delays; Efficiency; Efficient regulatory procedures; Inhouse testing or outsourcing; Logistical and operational issues; Logistics; Organisation of care; Turnaround time; Clinical interpretation; Necessity of specialized personnel, instrumentation, software, quality management; Need for optimized clinical workflows; Required expertise; Cancer screening; Early diagnosis; Monitoring; Monitoring cancer progression; Place in care pathway; Population wide implementation of testing; Position in care pathway; Position molecular testing in care pathway; Standardization of testing; Surveillance; Artificial intelligence; Breakthroughs in technology; Limited tissue material; Limited tumour samples; Liquid biopsy; Machine learning; Choosing between gene-panel and comprehensive approaches; Circulating tumour cells; Cytology samples; DNA input requirement; DNA yield; Quality assurance; Fresh frozen biopsies; Invasiveness biopsy; IT support; Optimization of tissue acquisition; Sample collection; Single gene tests; Targeted gene panels; Tissue optimization; Tissue preservation protocols; Tissue requirements; Using circulating cell-free DNA to monitor personalized cancer therapy; Technological / scientific advancements. |
| **Reimbursement (n=22)** | Clinical utility; Affordability of testing and treatments; Affordable testing; Cost; Cost-effectiveness; Economic impacts; Decreased costs; Future directions NGS; NGS; No biopsy costs / adverse events; Reproducibility and costs of microarray platforms; Test affordability; Treatment selection; WES; Disconnect between the funding of drugs and the related biomarker test; Financing; Funding; Funding from industry; Funding of tests and treatments; National strategy; Inconsistent reimbursement for molecular diagnostics; Reimbursement. |
| **Social (n=20)** | ELSI; Data sharing; Data storage; Workload of involved specialist; Education; Lack of knowledge; Specialist knowledge; Privacy concerns; Patient expectations; Attitude of pathologists to and level of involvement; Unsolicited findings; Clinical trial ethics; Population health impacts; Informed consent and patient education; Invasiveness biopsy; Patient and physician goals; Patient-reported outcomes; Quality of life; Resistance testing; Adoption. |
| **Market access (n=15)** | Approved treatments; Affordable testing and therapies; Access to drugs; Access to innovative drugs; Challenge of implementing targeted therapies; Lack of novel therapies; Access to testing; Novel NGS technique; The difficulties in developing novel molecularly targeted agents; Suboptimal drug development; Off-label drug use; Off-label treatment; Reduction in off-label targeted therapy costs; Market factors; Regulatory environment. |
| *, Number of factors that were originally extracted from literature before they were summarized under common headers.  NSCLC, Non-small cell lung cancer; DNA, deoxyribonucleic acid; IT, information technology; NGS, next-generation sequencing; WES, whole exome sequencing; ELSI, ethical, legal and social implications. | |

# Appendix III. Scenarios

**Explanation**

You will be first shown a status quo; the current situation from which the scenarios deviate. Following the status quo, you will be presented with nine scenarios. **The time horizon of each scenario is five years.**

**How are the scenarios structured?**
Each scenario starts with a possible future development, followed by potential consequences of that future development.

**Which questions will you ask about scenarios?**
We will ask you a series of questions about each scenario. The first question is always about the likelihood that that specific future development may occur. The following questions are related to the likelihood that the consequences of the future development may occur. Finally, we ask you to assess the likelihood that the overall scenario may occur. 

**Which inputs do I need to provide?**
We ask you to provide us with your personal judgements of three values:

1. The most likely probability (M) or modus that something may occur;
2. The lowest plausible bound (L); it should be extremely unlikely that the real probability is below this number;
3. The highest plausible bound (U); it should be extremely unlikely that the real probability is above this number.

Each of these values should be between 0 and 100.


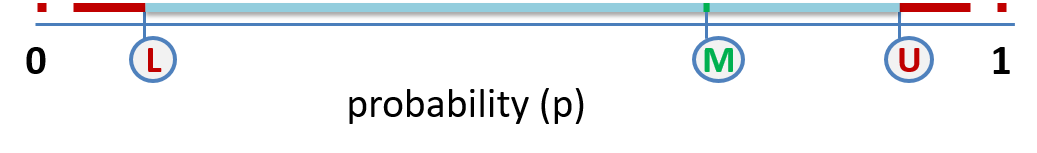


**Status quo**

The status quo represents the current situation and provides you with more context. Keep in mind that the scenarios are deviations from the status quo. The status quo is primarily based on available literature. In cases where no literature is available, estimates are used.

**Organization**
- WGS is **organized centrally**: one central facility conducts WGS for all hospitals
- **50% of the hospitals** that treat patients with advanced NSCLC offer WGS to their patients

**Clinical**
- Only NSCLC patients in stage IIIB and IV are eligibile for WGS
- Types of **standard diagnostic** tests: FISH, IHC, real-time PCR, Sanger sequencing, and NGS *[1]*
- Probability that an actionable target is found with **standard diagnostics** for on-label:

1. Targeted therapy: 8% *[2]*
2. Immunotherapy: 20% *[3]*

- Probability that an actionable target is found with **WGS** for:

1. Targeted therapy: 8%
2. Immunotherapy: 0%

- Probability that patients diagnosed with **standard diagnostics** have a treatment response to:

1. Chemotherapy: 30%*[4]*
2. Targeted therapy: 5% *[2]*
3. Immunotherapy: 7% *[3]*

​- ​​​Off-label drug prescription of targeted therapy is not allowed outside of clinical trials.

**Costs**
- Average costs of **standard diagnostics** per patients: €400,-
- Average costs of **WGS** per patient: €4500,-

**Technical**
- Turnaround time **standard diagnostics**: 1 week 
- Turnaround time **WGS**: 4 weeks
- WGS results for all patients need to be interpreted by a **molecular tumor board**
- Probability  that a tissue biopsy contains sufficient tumour cells to initiate diagnostic tests (especially for NGS, WGS): 60%
- Probability that **standard diagnostic** tests are successfully, resulting in useable information on which treatment selection can be made: 80%
- Probability that **WGS** is successfully, resulting in useable information on which treatment selection can be made: 80%

**Social**

- 80% of the physicians offer WGS to their patients
- 80% of the patients prefer WGS to other testing techniques

**Scenario 1**: Technological innovation in WGS has led to the development of a new WGS testing kit that is 50% cheaper in initial investment costs. Because of improvements in decision support in the new WGS device, interpretation by a molecular tumor board is only required for 5% of patients. This has decreased the average turnaround time of WGS to seven days. It enables all hospitals that treat advanced NSCLC to conduct WGS themselves and offer WGS to their patients.

What is the probability that a WGS testing kit that is 50% cheaper in initial investment costs will be developed within five years?

**Most likely probability (in%): lower plausible limit (in%): upper plausible limit (in%):**

What is the probability that, because of improvement in decision support, interpretation by a molecular tumor board is only required for 5% of the patients?

**Most likely probability (in%): lower plausible limit (in%): upper plausible limit (in%):**

What is the probability that, because interpretation by an MTB is only required for 5% of the patients, the average turnaround time of WGS will be reduced to seven days?

**Most likely probability (in%): lower plausible limit (in%): upper plausible limit (in%):**

What is the probability that this given overall scenario will take place in the next five years?

**Most likely probability (in%): lower plausible limit (in%): upper plausible limit (in%):**

**Scenario 2**: A new actionable biomarker has been identified that predicts response to immunotherapy. It is prevalent in 20% of patients with advanced lung cancer. Only WGS can detect this biomarker. The clinical utility of WGS has increased. Approximately 90%% of physicians are convinced of the value of WGS and thus offer WGS to all their patients with advanced NSCLC. Approximately 90% of patients with advanced NSCLC are also convinced of the value of WGS and thus prefer WGS to other molecular diagnostics.

What is the probability that WGS is the only testing technique that can identify a new biomarker for the next five years?

**Most likely probability (in%): lower plausible limit (in%): upper plausible limit (in%):**

What is the probability that a new biomarker identifies approximately 20% of the patients with advanced NSCLC as responsive to immunotherapy?

**Most likely probability (in%): lower plausible limit (in%): upper plausible limit (in%):**

What is the probability that, because of the increased clinical utility of WGS, a large majority of physicians will offer WGS to all their patients?

**Most likely probability (in%): lower plausible limit (in%): upper plausible limit (in%):**

What is the probability that, because of the increased clinical utility of WGS, a large majority of patients with advanced NSCLC prefer WGS to other molecular diagnostics?

**Most likely probability (in%): lower plausible limit (in%): upper plausible limit (in%):**

What is the probability that this given overall scenario will take place in the next five years?

**Most likely probability (in%): lower plausible limit (in%): upper plausible limit (in%):**

**Scenario 3:** WGS is organized completely centralized; one central facility that conducts all WGS for all hospitals. Due to economies of scale, this has led to large reductions in costs and turnaround time. The cost of WGS decreased to 1000 euro per patient. The average turnaround time decreased to five days. Because of the lower costs and a shorter turnaround time of WGS, all hospitals that treat patients with advanced lung cancer have adopted WGS.

What is the probability that centralizing the organisation of WGS leads to large reductions in the costs and turnaround time of WGS?

**Most likely probability (in%): lower plausible limit (in%): upper plausible limit (in%):**

What is the probability that the cost of WGS will decrease to 1000 euro per patient with cancer in this given scenario?

**Most likely probability (in%): lower plausible limit (in%): upper plausible limit (in%):**

What is the probability that the average turnaround time of WGS will decrease to five days in this given scenario?

**Most likely probability (in%): lower plausible limit (in%): upper plausible limit (in%):**

What is the probability that, because of lower cost and shorter turnaround time of WGS, all hospitals that treat patients with advanced NSCLC will adopt WGS?

**Most likely probability (in%): lower plausible limit (in%): upper plausible limit (in%):**

What is the probability that this given overall scenario will take place in the next five years?

**Most likely probability (in%): lower plausible limit (in%): upper plausible limit (in%):**

**Scenario 4:** WGS will become available as a diagnostic tool for NSCLC in clinical practice. The probability that WGS detects an actionable target for which targeted therapies are available is 12%. The average turnaround time is slightly reduced to 14 days. The costs of WGS are decreased to 3000 euro per patient.

What is the probability that WGS becomes available as a standard diagnostic tool for advanced NSCLC in clinical practice?

**Most likely probability (in%): lower plausible limit (in%): upper plausible limit (in%):**

What is the probability that WGS will detect an actionable target for which targeted therapies are available in 12% of the cases?

**Most likely probability (in%): lower plausible limit (in%): upper plausible limit (in%):**

What is the probability that the turnaround time of WGS is on average 14 days?

**Most likely probability (in%): lower plausible limit (in%): upper plausible limit (in%):**

What is the probability that the costs of WGS are reduced to 3000 euro per patient?

**Most likely probability (in%): lower plausible limit (in%): upper plausible limit (in%):**

What is the probability that WGS will be used as diagnostic tool instead of standard diagnostics, given this scenario?

**Most likely probability (in%): lower plausible limit (in%): upper plausible limit (in%):**

What is the probability that this given overall scenario will take place in the next five years?

**Most likely probability (in%): lower plausible limit (in%): upper plausible limit (in%):**

**Scenario 5:** A new liquid NGS panel ‘X’ will enter the market which provides only information about actionable targets that are needed for treatment selection. The probability that NGS panel ‘X’ detects an actionable target for which treatment is available is equal to that of standard diagnostics. For this new NGS panel ‘X’, less invasive and easy to obtain liquid biopsies can be used. The average turnaround time for this NGS panel ‘X’ is two days. The costs of NGS panel ‘X’ are 300 euro per patient.

What is the probability that a new liquid NGS panel ‘X’ will enter the market?

**Most likely probability (in%): lower plausible limit (in%): upper plausible limit (in%):**

What is the probability that NGS panel ‘X’ will detect an actionable target in 8% of the cases?

**Most likely probability (in%): lower plausible limit (in%): upper plausible limit (in%):**

What is the probability that less invasive and easy to obtain liquid biopsies can be used by NGS panel ‘X’?

**Most likely probability (in%): lower plausible limit (in%): upper plausible limit (in%):**

What is the probability that the turnaround time of NGS panel ‘X’ is on average two days?

**Most likely probability (in%): lower plausible limit (in%): upper plausible limit (in%):**

What is the probability that the costs of NGS panel ‘X’ are 300 euro per patient?

**Most likely probability (in%): lower plausible limit (in%): upper plausible limit (in%):**

What is the probability that the new liquid NGS panel will be used instead of WGS in this given scenario?

**Most likely probability (in%): lower plausible limit (in%): upper plausible limit (in%):**

What is the probability that this given overall scenario will take place in the next five years?

**Most likely probability (in%): lower plausible limit (in%): upper plausible limit (in%):**

**Scenario 6:** New technical innovations have resulted in better performance in cancer diagnostics. Tissue samples are still needed for WGS but due to new technologies there is a 80% probability that they contain enough tumour cells to initiate WGS. Another technical improvement is that there is a 95% probability that the sequencing process of WGS succeeds, resulting in useable information on which treatment selection can be made. Because of these low failure rates, more than 80% of the patients eligible for WGS can be sequenced and thus potentially receive better treatment. However, these new technologies come at a price and therefore, costs for sequencing one patients remain 4500 euro.

What is the probability that new technical innovations improve the success rate of taking tissue biopsies and the sequencing process of WGS?

**Most likely probability (in%): lower plausible limit (in%): upper plausible limit (in%):**

What is the probability that tissue biopsies have a 80% probability to be successfully taken in this given scenario?

**Most likely probability (in%): lower plausible limit (in%): upper plausible limit (in%):**

What is the probability that WGS has a 95% probability to be successfully sequenced in this given scenario?

**Most likely probability (in%): lower plausible limit (in%): upper plausible limit (in%):**

What is the probability that more than 80% of the patients can be sequenced and thus potentially receive better treatment in this given scenario?

**Most likely probability (in%): lower plausible limit (in%): upper plausible limit (in%):**

What is the probability that these new technologies are expensive and keep the costs of WGS per patient fixed at 4500 euros in this given scenario?

**Most likely probability (in%): lower plausible limit (in%): upper plausible limit (in%):**

What is the probability that this given overall scenario will take place in the next five years?

**Most likely probability (in%): lower plausible limit (in%): upper plausible limit (in%):**

**Scenario 7:** Research on WGS data results in the discovery of new molecular targets. As a result, new targeted therapies will be approved for these new targets. These new targets can only be detected with WGS, and therefore, the clinical utility has risen. The probability that an actionable target is found by WGS on which targeted therapies are indicated is increased to 20%. Because of this increase in actionable targets, 90% of the physicians prefer using WGS as molecular diagnostics. Because of this increase in actionable targets, 90% of patients prefer to receive WGS as molecular diagnostics.

What is the probability that research on WGS data results in the discovery of new molecular targets and as a result, new targeted therapies will be approved for these new targets?

**Most likely probability (in%): lower plausible limit (in%): upper plausible limit (in%):**

What is the probability that these new actionable targets can only be detected by WGS and therefore the clinical utility rises?

**Most likely probability (in%): lower plausible limit (in%): upper plausible limit (in%):**

What is the probability that WGS will detect an actionable target in 20% of the cases for which targeted therapies are available, given this scenario?

**Most likely probability (in%): lower plausible limit (in%): upper plausible limit (in%):**

What is the probability that physicians’ preference for WGS as molecular diagnostics increases to 90%, given this scenario?

**Most likely probability (in%): lower plausible limit (in%): upper plausible limit (in%):**

What is the probability that patients’ preference for WGS as molecular diagnostics increases to 90%, given this scenario?

**Most likely probability (in%): lower plausible limit (in%): upper plausible limit (in%):**

What is the probability that this given overall scenario will take place in the next five years?

**Most likely probability (in%): lower plausible limit (in%): upper plausible limit (in%):**

**Scenario 8:** Research on WGS data provides evidence about the effectiveness of targeted therapies indicated for other tumour types for mutations that are found in patients with advanced NSCLC. For this reason, off-label drug prescription in this patient population is allowed only for actionable targets detected with WGS. As a result, the clinical utility of WGS has risen. The probability that WGS detects an actionable target for off-label targeted therapies is 5%. Due to the possibility of receiving off-label targeted therapy when no on-label drugs are available, 95% of physicians prefer to use WGS as molecular diagnostics. Due to the possibility of receiving off-label targeted therapy when no on-label drugs are available, all patients prefer to receive WGS as molecular diagnostics.

What is the probability that research on WGS data provides evidence for effective off-label drug use and as a result off-label drug use will be allowed?

**Most likely probability (in%): lower plausible limit (in%): upper plausible limit (in%):**

What is the probability that off-label drug prescription is allowed only on actionable targets that are found with WGS?

**Most likely probability (in%): lower plausible limit (in%): upper plausible limit (in%):**

What is the probability that WGS detects an actionable target for off-label targeted therapies in 5% of the cases, given this scenario?

**Most likely probability (in%): lower plausible limit (in%): upper plausible limit (in%):**

What is the probability that physicians’ preference for WGS as molecular diagnostics increases to 95%, given this scenario?

**Most likely probability (in%): lower plausible limit (in%): upper plausible limit (in%):**

What is the probability that all patients prefer to receive WGS as molecular diagnostics, given this scenario?

**Most likely probability (in%): lower plausible limit (in%): upper plausible limit (in%):**

What is the probability that this given overall scenario will take place in the next five years?

**Most likely probability (in%): lower plausible limit (in%): upper plausible limit (in%):**

**Scenario 9:** Patients with a particular actionable target for targeted therapy identified with WGS have a higher probability to respond to treatment than patients with the same target identified with standard diagnostics. The probability for having a treatment response to targeted therapy is increased to 10% for a patient with an actionable target detected with WGS. Since WGS detects biomarkers that are better predictors for treatment response, its clinical utility has risen. For this reason, all physicians prefer to use WGS as molecular diagnostics. For this reason, all patients prefer to receive WGS as molecular diagnostics.

What is the probability that patients with a particular actionable target identified with WGS have a better treatment response than patients with the same target identified with standard diagnostics?

**Most likely probability (in%): lower plausible limit (in%): upper plausible limit (in%):**

What is the probability that the probability for having a treatment response to targeted therapy is increased to 10% for a patient with an actionable target detected with WGS?

**Most likely probability (in%): lower plausible limit (in%): upper plausible limit (in%):**

What is the probability that the clinical utility of WGS has risen since it detects biomarkers that are better predictors for treatment response?

**Most likely probability (in%): lower plausible limit (in%): upper plausible limit (in%):**

What is the probability that all physicians prefer to use WGS as molecular diagnostics, given this scenario?

**Most likely probability (in%): lower plausible limit (in%): upper plausible limit (in%):**

What is the probability that all patients prefer to receive WGS as molecular diagnostics, given this scenario?

**Most likely probability (in%): lower plausible limit (in%): upper plausible limit (in%):**

What is the probability that this given overall scenario will take place in the next five years?

**Most likely probability (in%): lower plausible limit (in%): upper plausible limit (in%):**

**References**

*[1]      Planchard D, Popat S, Kerr K, Novello S, Smit EF, Faivre-Finn C, et al. Metastatic non-small cell lung cancer: ESMO Clinical Practice Guidelines for diagnosis, treatment and follow-up. Annals of oncology : official journal of the European Society for Medical Oncology. 2018;29(Supplement_4):iv192-iv237.
[2]      Marquart J, Chen EY, Prasad V. Estimation of the Percentage of US Patients With Cancer Who Benefit From Genome-Driven Oncology. JAMA oncology. 2018;4(8):1093-8.
[3]      Haslam A, Prasad V. Estimation of the Percentage of US Patients With Cancer Who Are Eligible for and Respond to Checkpoint Inhibitor Immunotherapy Drugs. JAMA network open. 2019;2(5):e192535.
[4]      Ardizzoni A, Boni L, Tiseo M, Fossella FV, Schiller JH, Paesmans M, et al. Cisplatin- versus carboplatin-based chemotherapy in first-line treatment of advanced non-small-cell lung cancer: an individual patient data meta-analysis. Journal of the National Cancer Institute. 2007;99(11):847-57.*

# Appendix IV. Characteristics of the experts that responded to the scenario survey

Table A3. Characteristics of the experts that responded to the scenario survey

| Expert # | **Field(s) of expertise** | **Nationality** |
| --- | --- | --- |
| 1 | Genetics | the Netherlands |
| 2 | Genetics / informatics | the Netherlands |
| 3 | Genetics / informatics / oncology | the Netherlands |
| 4 | Genetics / oncology / pathology | the Netherlands |
| 5 | Genetics / Pathology | the Netherlands |
| 6 | Health economics / health technology assessment | the Netherlands |
| 7 | Lung cancer / Pulmonary disease | the Netherlands |
| 8 | Oncology | the Netherlands |
| 9 | Oncology | the Netherlands |
| 10 | Oncology | the Netherlands |
| 11 | Pathology | the Netherlands |
| 12 | - | the Netherlands |
| 13 | - | the Netherlands |
| 14 | - | the Netherlands |
| 15 | - | the Netherlands |
| 16 | - | the Netherlands |
| 17 | Genetics / Oncology | Singapore |
| 18 | - | Australia |
| 19 | - | Denmark |
| ‘-‘, not specified. | | |

# Appendix V. Linear pools of individual distributions


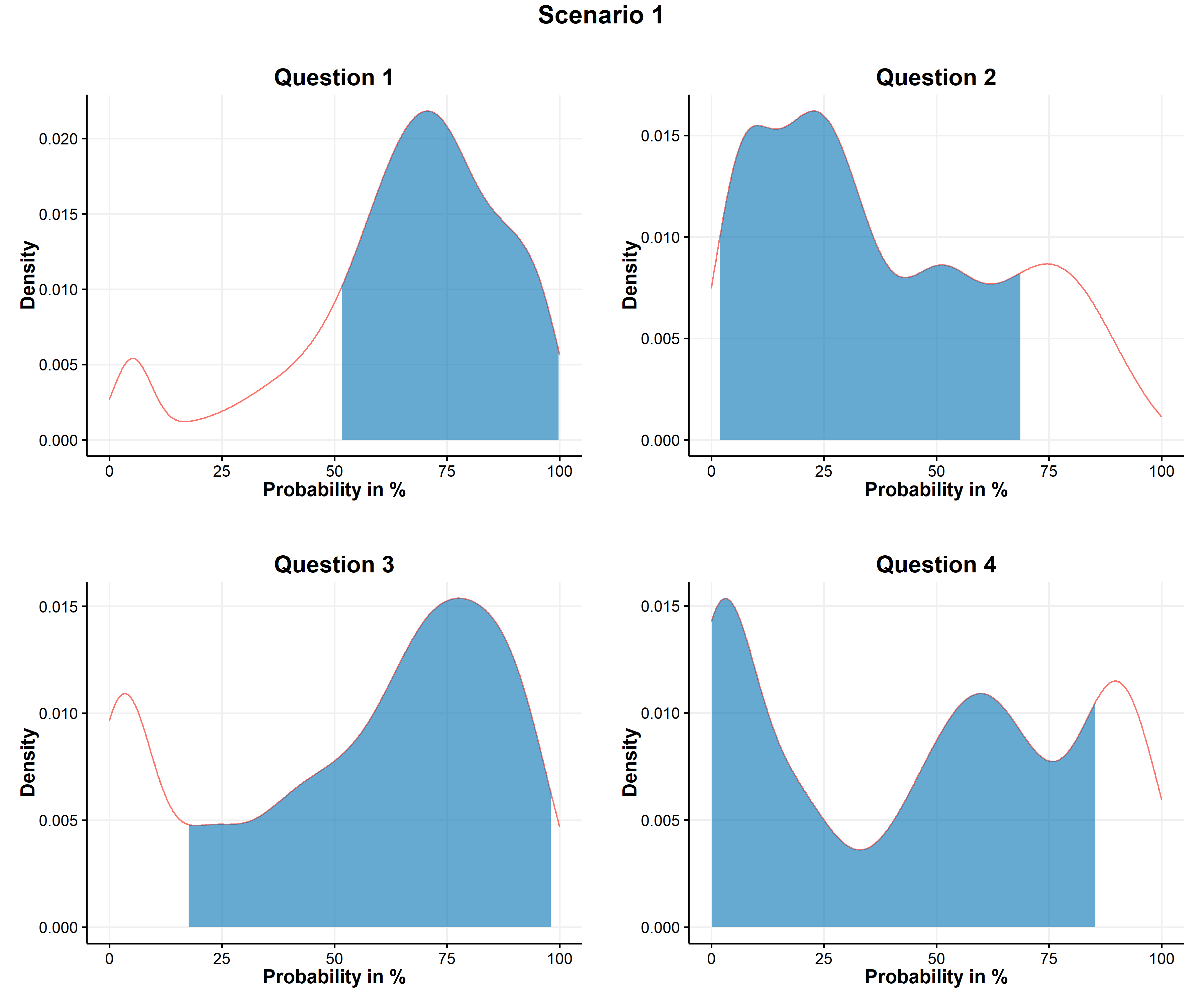


Figure A2. Linear pools of individual PERT distributions of scenario 1.


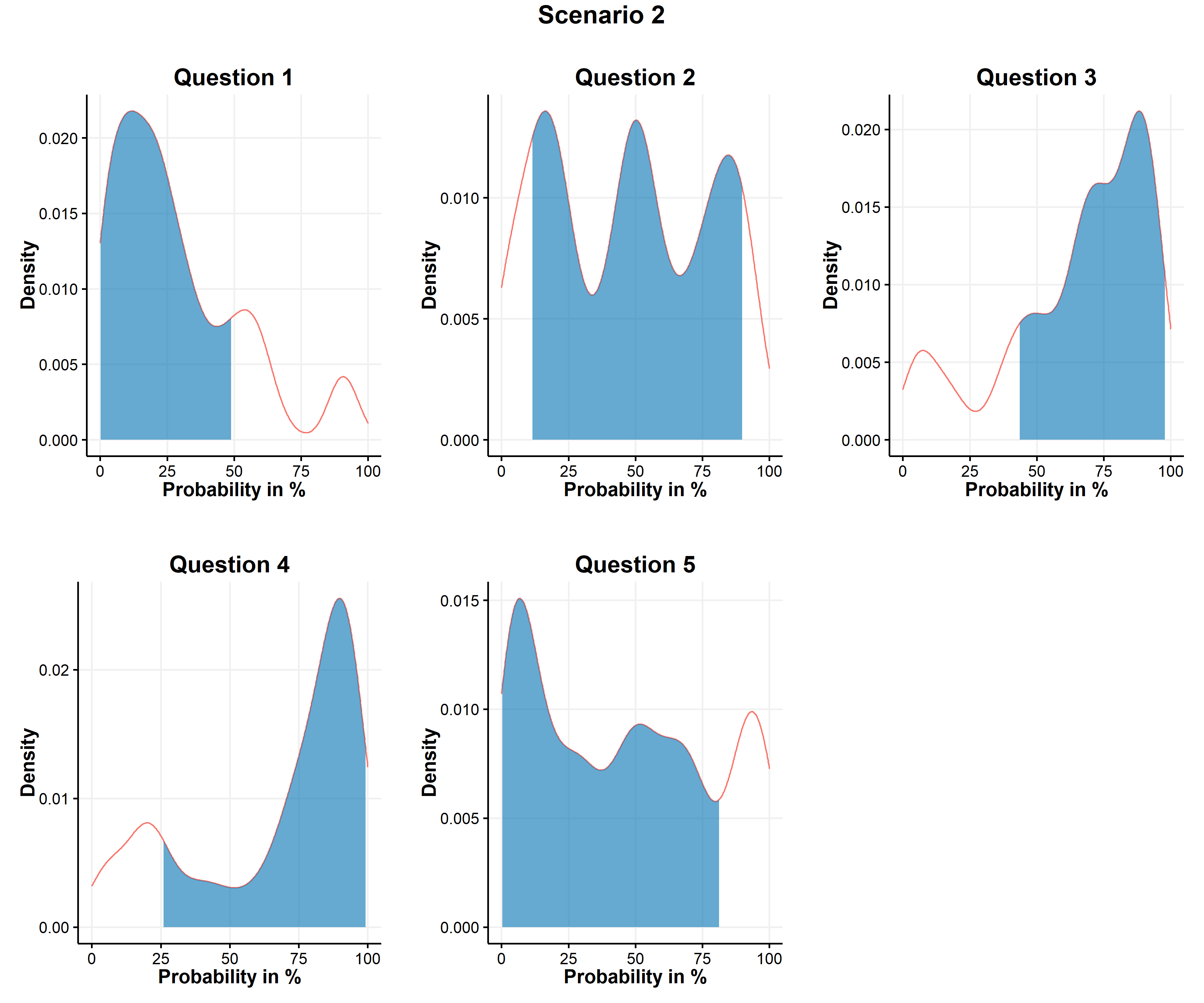
 Figure A3. Linear pools of individual PERT distributions of scenario 2.


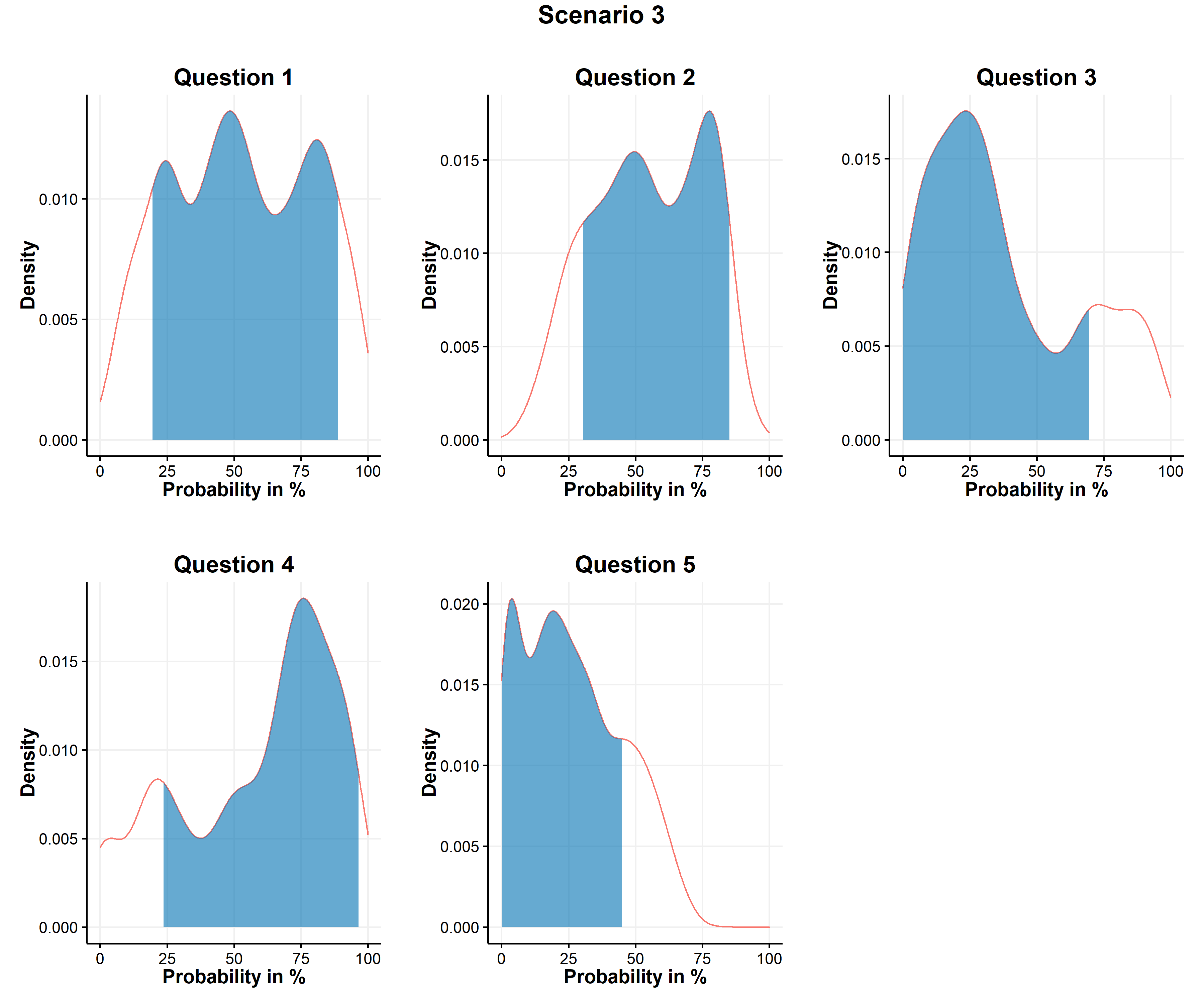
 Figure A4. Linear pools of individual PERT distributions of scenario 3.


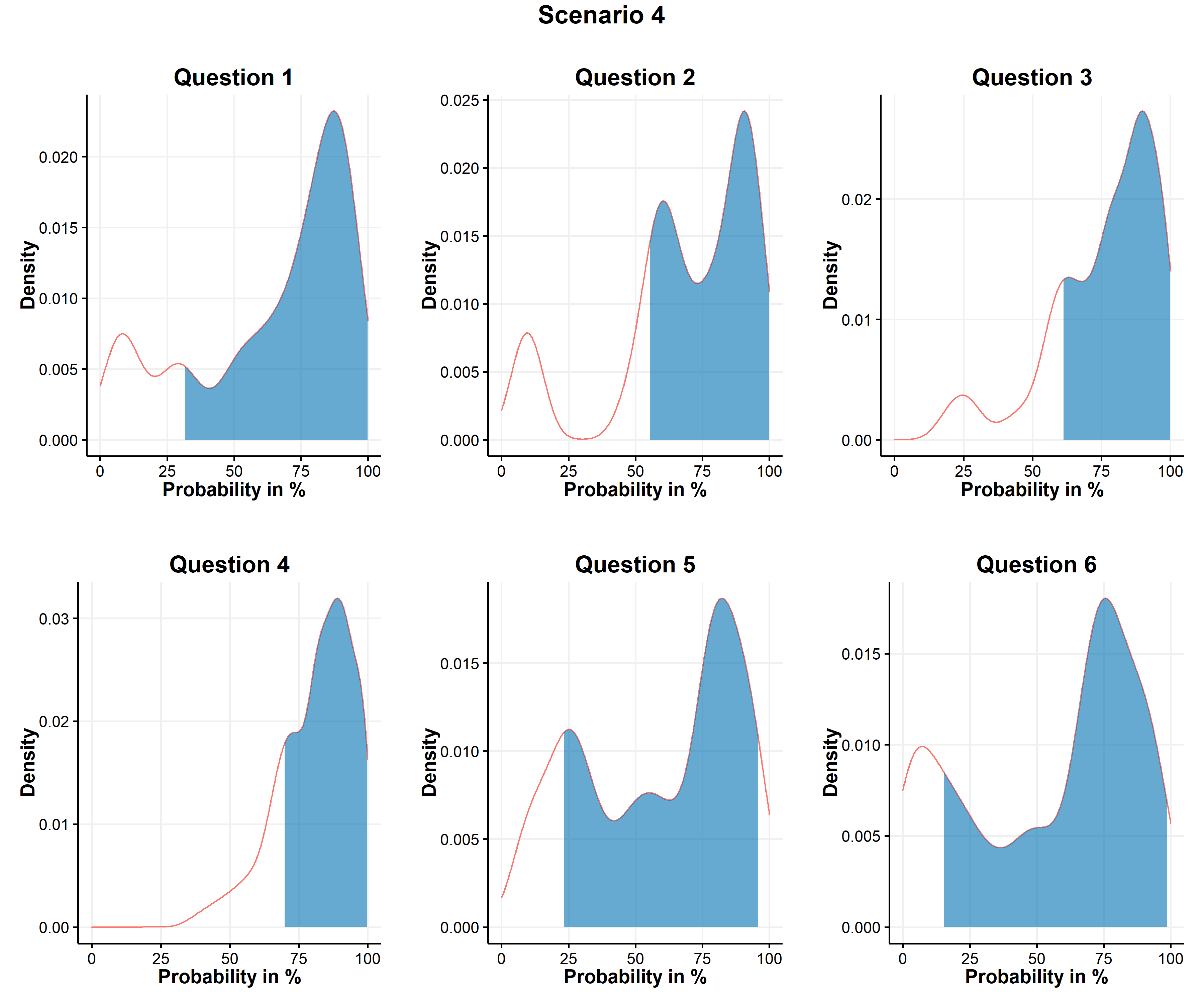
 Figure A5. Linear pools of individual PERT distributions of scenario 4.


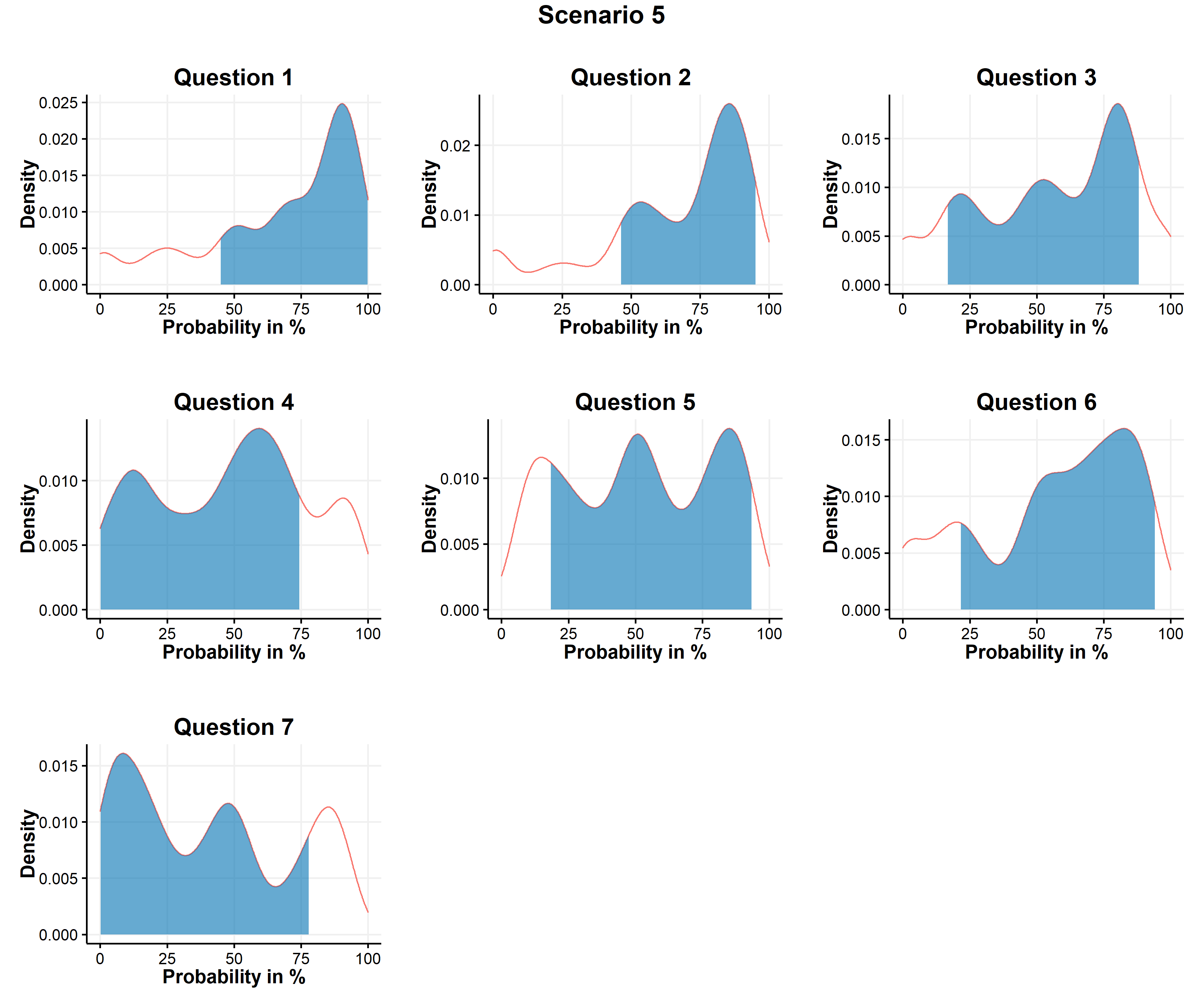
 Figure A6. Linear pools of individual PERT distributions of scenario 5.


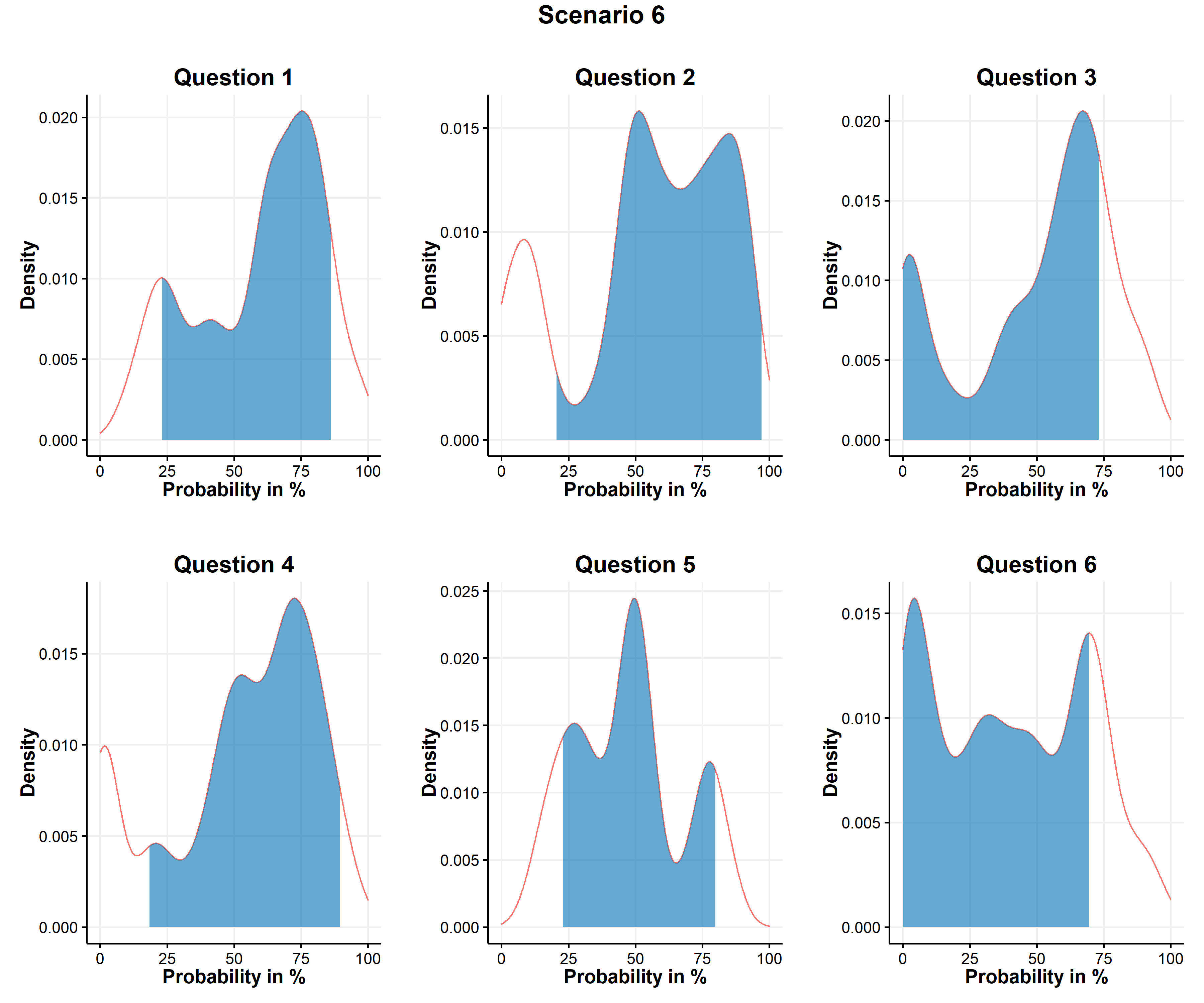
 Figure 7. Linear pools of individual PERT distributions of scenario 6.


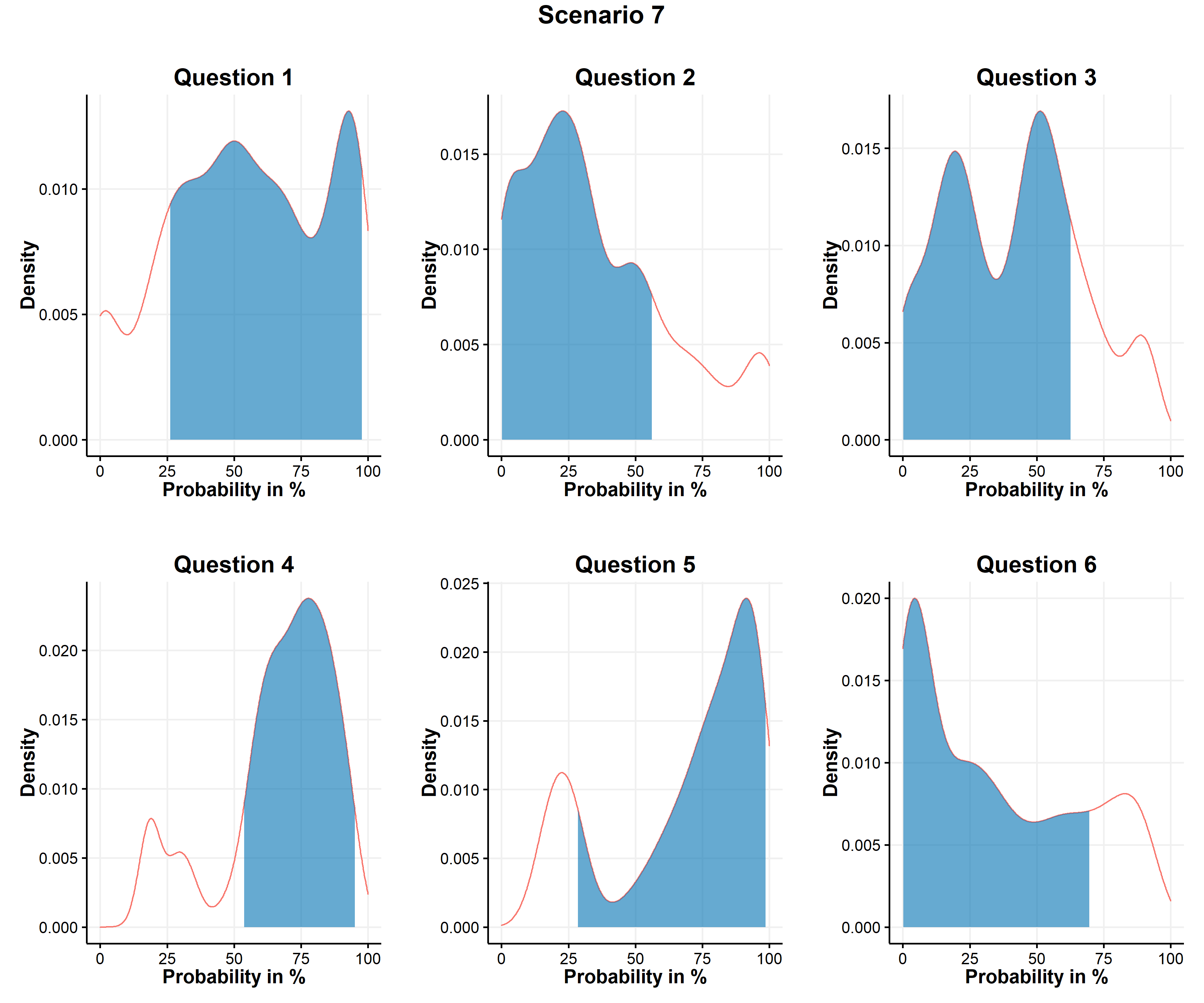
 Figure A8. Linear pools of individual PERT distributions of scenario 7.


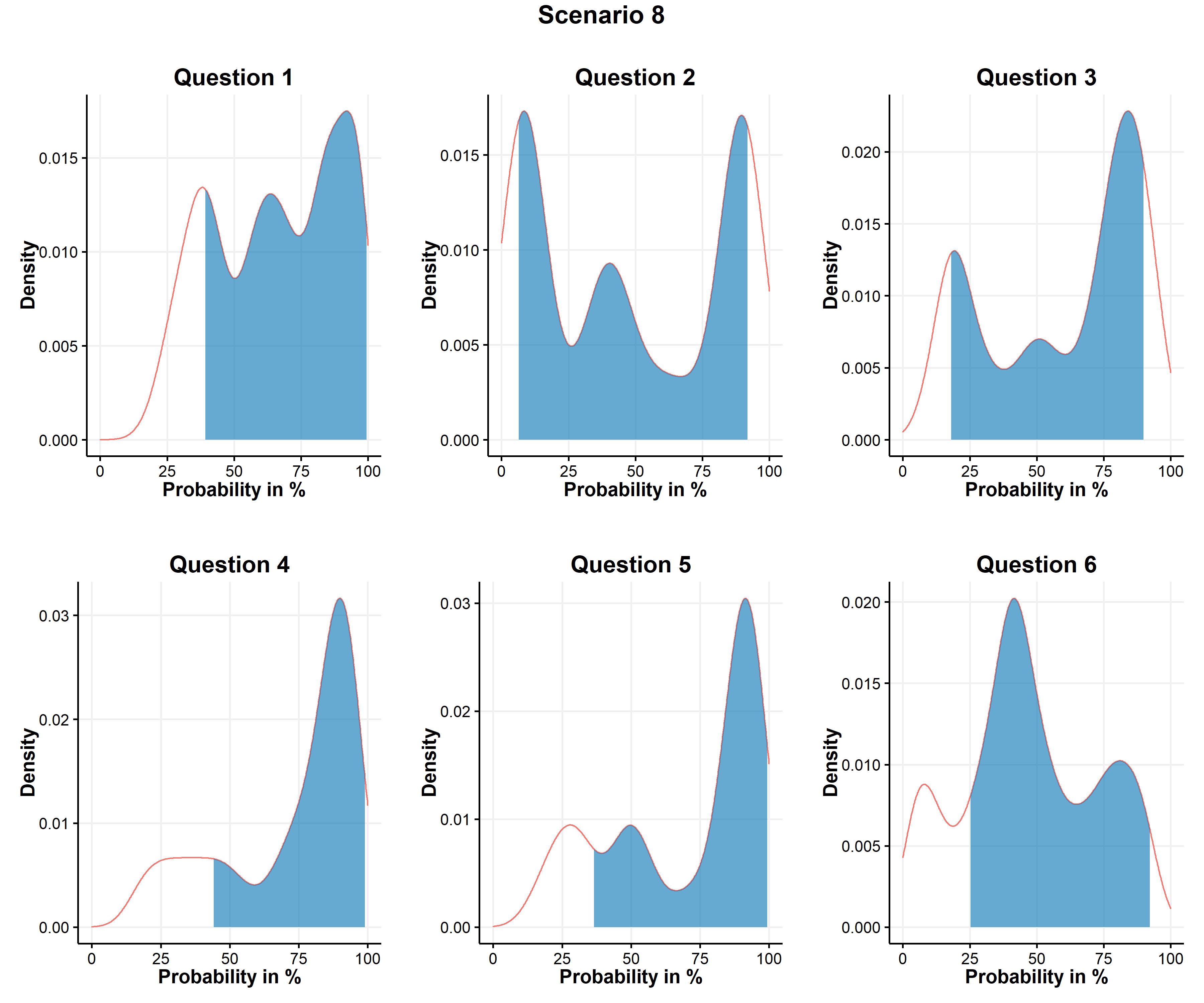
 Figure A9. Linear pools of individual PERT distributions of scenario 8.


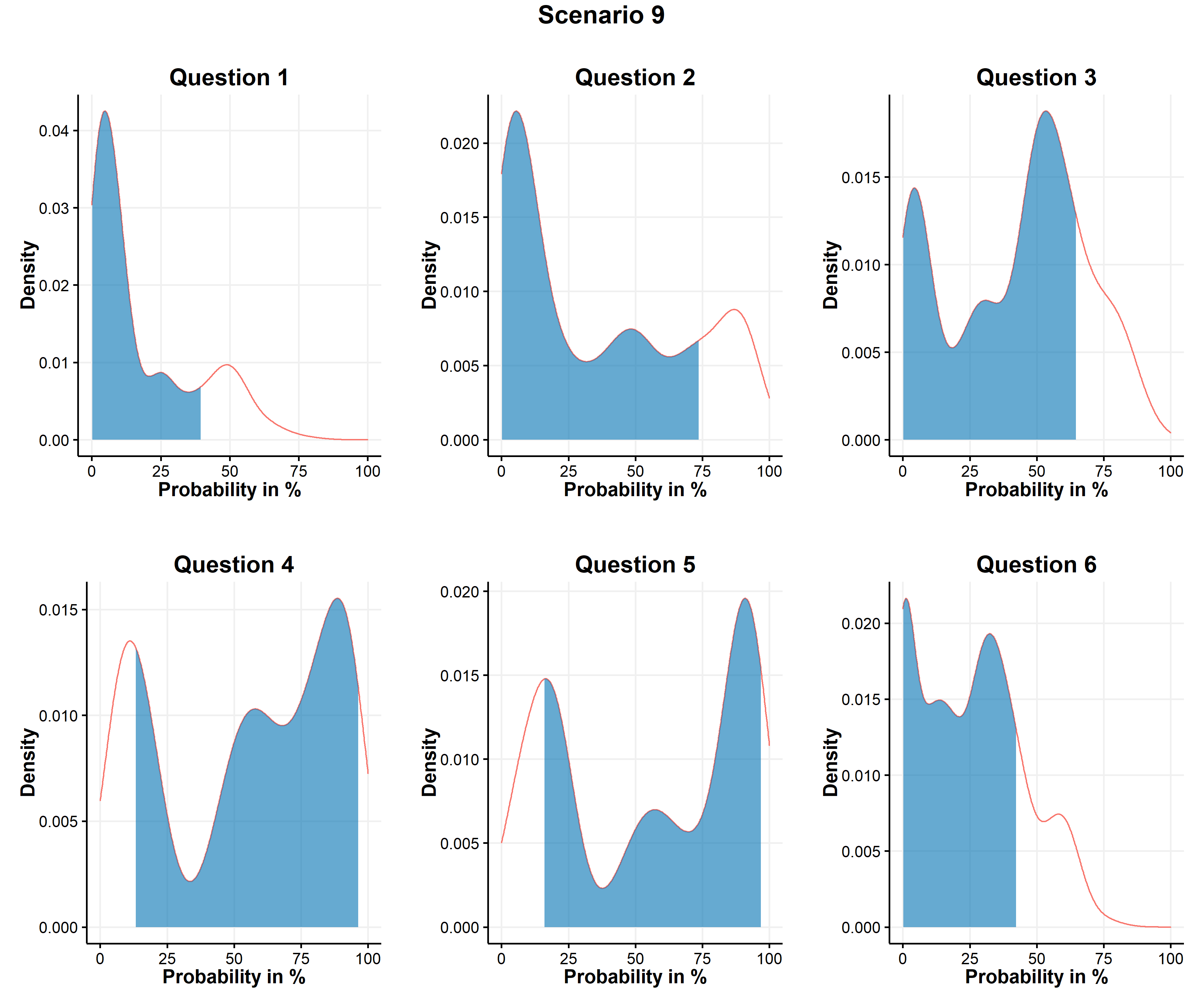
 Figure A10. Linear pools of individual PERT distributions of scenario 9.
